# Attenuated Bacteria as Immunotherapeutic Tools for Cancer Treatment

**DOI:** 10.3389/fonc.2018.00136

**Published:** 2018-05-01

**Authors:** Suneesh Kaimala, Ashraf Al-Sbiei, Otavio Cabral-Marques, Maria J. Fernandez-Cabezudo, Basel K. Al-Ramadi

**Affiliations:** ^1^Department of Medical Microbiology and Immunology, College of Medicine and Health Sciences, United Arab Emirates University, Al Ain, United Arab Emirates; ^2^Department of Biochemistry, College of Medicine and Health Sciences, United Arab Emirates University, Al Ain, United Arab Emirates; ^3^Center for Chronic Immunodeficiency (CCI), Medical Center-University of Freiburg, Faculty of Medicine, University of Freiburg, Freiburg, Germany

**Keywords:** bacterial therapy, attenuated *Salmonella*, cancer immunotherapy, tumor microenvironment, tumor-infiltrating leukocytes, myeloid-derived suppressor cells

## Abstract

The use of attenuated bacteria as cancer therapeutic tools has garnered increasing scientific interest over the past 10 years. This is largely due to the development of bacterial strains that maintain good anti-tumor efficacy, but with reduced potential to cause toxicities to the host. Because of its ability to replicate in viable as well as necrotic tissue, cancer therapy using attenuated strains of facultative anaerobic bacteria, such as *Salmonella*, has several advantages over standard treatment modalities, including chemotherapy and radiotherapy. Despite some findings suggesting that it may operate through a direct cytotoxic effect against cancer cells, there is accumulating evidence demonstrating that bacterial therapy acts by modulating cells of the immune system to counter the growth of the tumor. Herein, we review the experimental evidence underlying the success of bacterial immunotherapy against cancer and highlight the cellular and molecular alterations in the peripheral immune system and within the tumor microenvironment that have been reported following different forms of bacterial therapy. Our improved understanding of these mechanisms should greatly aid in the translational application of bacterial therapy to cancer patients.

The practice of using bacteria for cancer therapy dates back to the nineteenth century. In 1893, William Coley, a New York-based physician, prepared a filtered mixture of bacteria and bacterial lysates, composed of *Streptococcus pyogenes* and *Bacillus prodigiosus* (now called *Serratia marcescens*) and called it “Coley’s Toxin.” He found that, in some cases, the tumors regress when Coley’s toxin is injected into the tumors (1). Later on he developed a safe vaccine, a mixture of heat killed *S. pyrogenes* and *Seretia marcescenes*, to successfully treat sarcoma, carcinoma, lymphoma, melanoma, and myeloma (2). These procedures practiced by Coley formed the basis of the recent advances in the cancer immunotherapy using attenuated bacterial strains. Today, the most common species of bacteria being used as immunotherapeutic agents are *Clostridium novyi* (3, 4), *Listeria monocytogenes* (5, 6), and *Salmonella enterica* serovar Typhimurium (hereafter referred to as *Salmonella typhimurium*) (6–10). Infection of poorly antigenic tumors with facultative anaerobic bacteria is thought to increase their antigenicity. Bacterial infections also alter the function of different cellular components of the immune system, such as CD4^+^ and CD8^+^ T cells, myeloid-derived suppressor cells (MDSCs), regulatory T cells (Tregs), tumor-associated macrophages (TAMs), and their activation. Many conserved bacterial ligands are agonists for innate immune system receptors, such as toll-like receptors (TLR), and upon binding, initiate an intracellular signaling cascade leading to the production of proinflammatory cytokines. Moreover, there is evidence that some bacterial components, such as exotoxins, may initiate anti-tumor activities not only by indirect activation of the immune system, but also by their direct action on tumor cells (11, 12). In addition to their immunotherapeutic properties against diverse types of cancers, *Salmonella* and *Listeria* are also used as vectors for delivering immunogenic tumor antigens to the host. The use of bacteria for delivering tumor antigens has been reviewed in detail elsewhere (13, 14). Here, we aim to review the direct effects of bacterial immunotherapeutic agents on different cellular components of the immune system.

## Effects of Bacterial Therapy on Myeloid Cells

### Tumor-Associated Macrophages

As the tumor starts growing it gets enriched with different myeloid cell populations, such as MDSCs, tumor-associated neutrophils (TANs), TAMs, Tek tyrosine kinase receptor (TIE)-2-expressing monocytes, and tolerogenic dendritic cells (15, 16). Monocytes are recruited to sites of tumor growth in response to chemoattractants released by tumor cells, such as colony stimulating factor 1 and the chemokine C-C motif chemokine ligand 2 (CCL2) (17), where they differentiate into macrophages. Usually, these are alternatively activated cells involved in tissue repair, also known as M2-type macrophages (18), and they express immunosuppressive molecules, such as arginase 1 (Arg1) (19) and the cytokine IL-10 (20). M2 macrophages support the growth and malignancy of tumors by suppressing the host’s anti-tumor immune responses. In contrast, M1 macrophages, also known as classically activated or killer macrophages, express nitric oxide synthase (NOS_2_) and TNF-α and orchestrate protective anti-tumor immune responses (21, 22).

**Figure 1:**
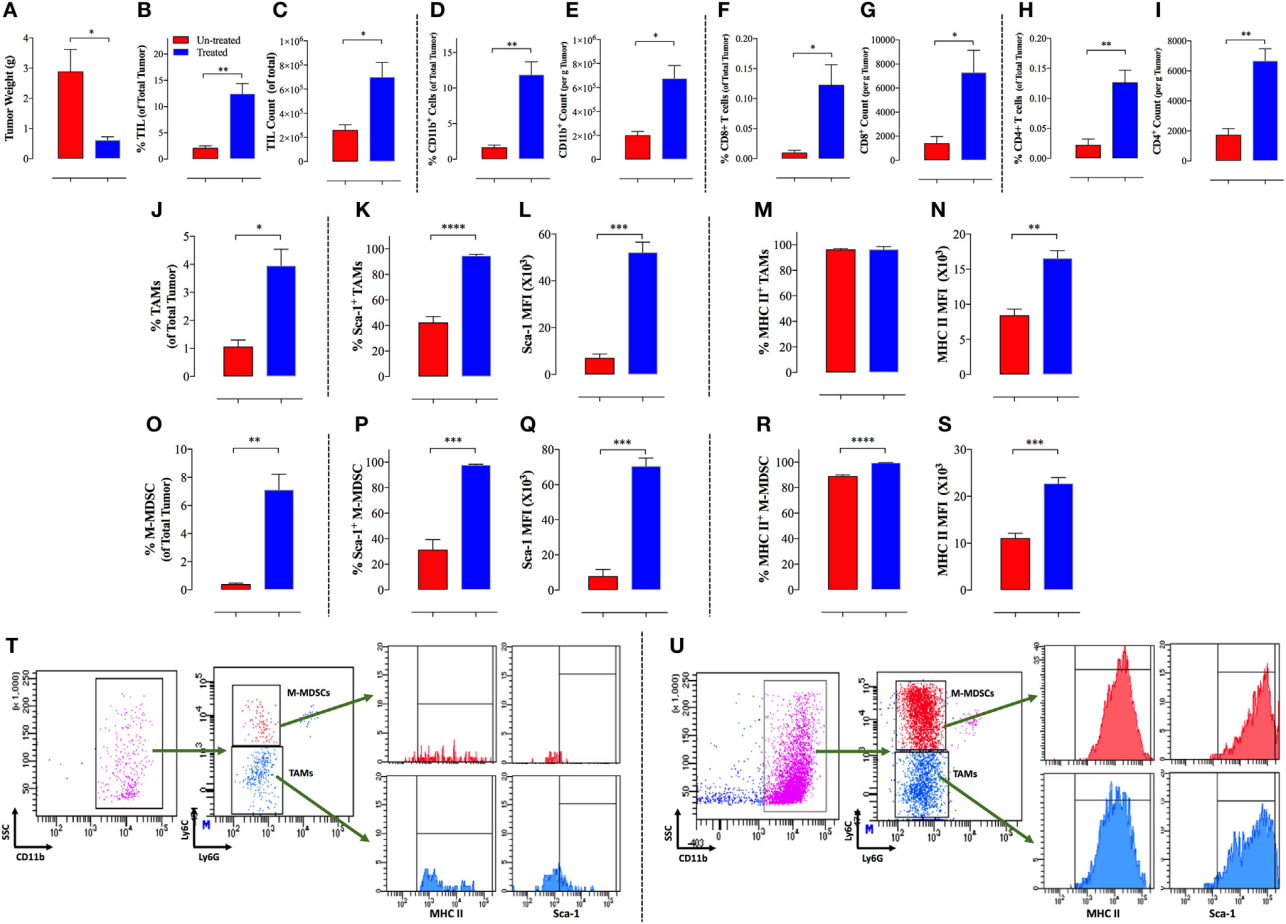
Increased immunogenicity of B16.F1 melanoma tumors following intraperitonial treatment with *Salmonella typhimurium* strain BRD509E. Decreased tumor weights **(A)** in *Salmonella*-treated mice correlates with increased percentage **(B)** and absolute counts **(C)** of tumor-infiltrating leukocytes (TIL) as elucidated by CD45 expression and analyzed by multi-color FACS analysis of total tumor cells. Increased cellular infiltration into tumor tissue is observed for CD11b^+^ myeloid cells **(D,E)**, CD8^+^ T cells **(F,G)**, and CD4^+^ T cells **(H,I)**. The increase in cellular infiltration is observed in terms of the percentage of indicated cells in the whole tumor **(D,F,H)** and in terms of absolute cell counts per gram of tumor tissue **(E,G,I)**. *Salmonella* treatment also enhanced infiltration and maturation of tumor-associated macrophages (TAMs) **(J–N)** and monocytic myeloid-derived suppressor cells (MDSCs) **(O–S)**. Percentage of CD11b^+^F4/80^+^Ly6G^−^Ly6C^−^ TAMs **(J)** and CD11b^+^Ly6G^−^Ly6C^+^ M-MDSCs **(O)** among total tumor cells. The proportion of both myeloid cell types was significantly higher in *Salmonella*-treated tumors than non-treated tumors. Percentage of Sca-1-positive and Sca-1 expression level shown as mean fluorescent intensity among TAMs **(K,L)** and MDSCs **(P,Q)**. Percentage of MHC class II-positive and MHC class II expression level among TAMs **(M,N)** and MDSCs **(R,S)**. **(T–U)** Representative FACS plots of the data highlighting the gating strategy for untreated **(T)** and *Salmonella*-treated tumors **(U)**. The gated CD11b^+^ cells are out of selected CD45^+^ cells. The analysis was carried out on day 12 post *Salmonella* administration. Asterisks indicate significant differences, *p < 0.05; **p < 0.01; ***p < 0.001; ****p < 0.0001.

We have recently reported that treatment of mice bearing B16.F1 melanoma with an attenuated strain of *S. typhimurium* led to maturation of intratumoral myeloid cells and diminished their suppressive capacity, enhancing the host’s anti-tumor immune responses, and eventually leading to tumor regression (23). Multi-color flow cytometric analysis of tumor-infiltrating leukocytes (TILs) revealed that *Salmonella* induced tumor inhibition (Figure 1A) results in increased immune responses in the tumor microenvironment. This fact is evidenced by the significantly elevated levels of TILs in the tumors of *Salmonella*-treated mice (Figures 1B,C) including both CD11b^+^ myeloid cells (Figures 1D,E) and CD4^+^ and CD8^+^ T cells (Figures 1F–I). Analysis of the different subpopulations of tumor-associated myeloid cells showed that *Salmonella* treatment led to a large increase in the proportion of cells characterized by being CD11b^+^/F4/80^+^/Ly6C^−^/Ly6G^−^, which are commonly known as TAMs (Figure 1J). Upon treatment with *Salmonella*, TAMs increase the expression of M1-type macrophage activation markers, such as the IFN-γ-dependent Sca-1 and MHC class II proteins (Figures 1K–N). Representative FACS plots of the data highlighting the gating strategy for untreated (Figure 1T) and *Salmonella*-treated tumors (Figure 1U) are also shown. This observation indicates that treatment with *Salmonella* skews the TAMs profile, reprogramming them toward proinflammatory functions and indicating the functional plasticity of M1/M2 macrophages. A similar shift in the phenotypic characteristics of macrophages was reported by Hong and coworkers upon intratumoral injection of a recombinant, attenuated *S. typhimurium* vaccine into Her2/Neu-expressing CT26 tumors (24). Following the injections, the phenotype of the splenic and intratumoral macrophage populations was shifted from an immature to mature-type expressing TNFα (24). Treatment of mice bearing an aggressive ID8-Defb29/Vegf-A ovarian carcinoma with an attenuated strain of *L. monocytogenes* (ΔactA/ΔinlB) also resulted in augmented infiltration of macrophages into the tumor and in a shift from their M2 to M1 profile (25). These macrophages displayed elevated phagocytic and tumoricidal activity. Moreover, TAMs in the peritoneal exudates of treated mice exhibited increased expression of the co-stimulatory molecules CD80 and CD86, increased gene expression of proinflammatory cytokines, and downregulated transcriptional activity of suppressive effector molecules (25).

### Dendritic Cells and TANs

Of note, it was recently reported that murine or human melanoma cell lines infected with pathogenic *L. monocytogenes* (LM^WT^), transformed these malignant melanocytes into professional antigen-presenting cells (APCs) with a phenotype and function analogous to those of skin DCs (5). These infected melanoma cells expressed phenotypic markers, such as CD11c, F4/80, MHCII, CD40, and CD83 similar to mature dendritic cells (5). The mechanisms explaining these phenotypic changes by invasive pathogens, such as *Salmonella*, consist in their capacity to stimulate TLR signaling pathways, which also enhance the expression of co-stimulatory molecules (e.g., CD86 and CD80) on APCs (26). Such events, enable APCs to strongly activate antigen-specific CD8 T cells and natural killer (NK) cells, which in turn, mediate tumor killing and regression. Most TLRs signal through myeloid differentiation primary response 88 (MyD88), an essential cytoplasmic adaptor protein that links triggering of TLRs and IL-1/IL-18 receptors with downstream activation of IL-1 receptor-associated kinases (IRAKs) and NF-κB (27). Treatment of MyD88-deficient mice carrying B16.F1 tumors with attenuated *Salmonella* was ineffective in regressing tumor growth, indicating that bacterial therapy of tumors is dependent on TLR-MyD88 signaling (23). These findings suggest that the anti-tumor effect of attenuated *Salmonella* is mediated through changes in intratumoral myeloid cells.

The effect of *Salmonella* immunotherapy on TANs has also been reported. Treatment of the mammary LM3 adenocarcinoma with a *Salmonella typhi* vaccine strain led to the activation and recruitment of neutrophils into the tumor site. These neutrophils were able to secrete TNFα, which in combination with IFNγ, exerted synergistic cytotoxic effects on endothelial and tumor cells within the tumor microenvironment (28). Recruitment of TANs into the tumor tissue was observed when a B-cell non-Hodgkin lymphoma was intratumorally injected with the attenuated *S. typhimurium* strain LVR01. This treatment elicited both local and systemic anti-tumoral immune responses, eventually leading to enhanced host survival (29). The exact cause of the phenotypic changes in myeloid cells during bacterial immunotherapy is not known. Such alterations in intratumoral myeloid cells have also been reported during cancer therapy using the parasitic protozoan *Toxoplasma gondii* (30, 31). It is likely that stimulation of TLR signaling by various bacterial components could account for these effects (32).

## Effects of Bacterial Therapy on Tumor-Associated Lymphoid Cells

The tumor microenvironment harbors lymphoid subpopulations, such as NK cells, B cells, CD4^+^ T cells, CD8^+^ T cells, and Tregs (15, 16). The lymphoid cells can be tumor promotive or suppressive in nature. The tumor microenvironment is a critical factor that influences the function of these lymphoid subpopulations in the tumor (33).

### NK Cells

Natural killer cells are prototypical innate lymphoid cells able to recognize and eradicate tumor cells without prior antigenic exposure. However, immunosubversion (i.e., when the tumor suppresses the host immune system) as well as immunoediting or immunoselection (i.e., outgrowth of poorly immunogenic tumor-cell variants) impairs NK cell responses to tumors (34, 35). We have previously shown that NK cells are readily activated by a recombinant *Salmonella* strain engineered to express IL-2 (36). Moreover, this recombinant strain exhibited superior anti-tumor activity against B16.F1 melanoma in a syngeneic tumor model, an activity that correlated with its capacity to induce a higher level of tumor cell killing *in vivo* (37). In addition, *Salmonella* strains expressing IFN-γ or TNF-α have been shown to induce highly effective immune-potentiating anti-microbial as well as anti-tumoral responses (38–41). The enhanced anti-tumoral capacity of an engineered IFN-γ-expressing *Salmonella* strain was dependent on the activation of NK cells (41). A recent study reported that treatment of non-Hodgkin lymphoma-bearing mice with attenuated *Salmonella* resulted in increased expression of chemokines, such as *Ccl2, Ccl3*, and *Ccl5*, which are involved in the recruitment of NK cells, and enhanced NK cell cytotoxic activity against target cells (42).

### CD4^+^ and CD8^+^ T Cells

The anti-tumor functions of CD4 T-cell depend upon their specific subset (Th1, Th2, or Th17 cells) (43). Th1 cells are anti-tumorigenic by virtue of either activating CD8 T cells (44) or directly killing tumor cells by secreting TNF-α and/or IFN-γ (45). Th1 cells also act against tumor development by licensing DCs and macrophages, e.g., increasing their antigen-presenting potential that enable T CD8^+^ cells to develop a strong cytotoxic activity (45). On the other hand, while Th2 cells are less relevant to cancer pathology, Th17 cells found within the tumor microenvironment facilitate tumor growth by the secretion of IL-17, a pro-angiogenic cytokine (46).

In the B16.F1 melanoma model, intraperitoneal treatment with attenuated *Salmonella* led to increased infiltration of both CD4^+^ and CD8^+^ T cells into the tumor (Figures 1F–I). Both T cell types were increased by >threefold in terms of percentage within total tumor cells and in their absolute counts per weight of tumor tissue. CCL5 and macrophage inflammatory protein 1 alpha, which are secreted by proinflammatory M1 macrophages, are known to recruit activated T cells into the tumor (47). Hence, this represents a mechanism by which activated macrophages induced by *Salmonella* treatment could be responsible for the enhanced T cell infiltration into tumors. Several studies have reported similar effects as a result of administration of attenuated bacteria in different models. When mice bearing EL4 lymphoma were immunized with *Salmonella typhi* by injecting the bacteria into the tumor and the draining lymph node areas, the leukocyte populations in the tumor draining lymph nodes were expanded and tumor growth was significantly decreased (48). The tumors in treated mice contained significantly decreased levels of IL-10 and this was accompanied by a reduction in the mitotic index of tumors, a delayed development of palpable lymph node metastases and, most importantly, increased survival compared to untreated mice. In another study, an enhancement of tumor-infiltrating activated CD8 T cells was observed following treatment of mice carrying A20 lymphoma with the attenuated *S. typhimurium* LVR-01 strain, which consequently led to a reduction in tumor growth (29). When the vaccine was supplemented with IL-2, there was an increased infiltration of CD4 and CD8 T cells in the tumors. The combined treatment resulted in better control of tumor growth and improved animal survival significantly over the *Salmonella* vaccine alone treatment (49). This effect is similar to our previously reported findings demonstrating the superior anti-tumor activity of an IL2-expressing *Salmonella* strain in the melanoma model (37). Thus, treatment of the tumors with *Salmonella* alone or in combination with interleukins potentiates the tumoricidal activity of tumor-infiltrating lymphocytes. Moreover, there is direct evidence that cytokine-expressing *Salmonella* strains can strongly modulate macrophage activation and function (40). Using CD4 and CD8 T cell-deficient mice, Lee and coworkers showed that *Salmonella* treatment of Lewis lung carcinoma (LL2)-bearing mice was relatively less efficient in the absence of T cells (34–42% inhibition in tumor growth compared to 50% in WT mice) (50). Systemic treatment with *Salmonella* induced a Th1 inflammatory response at the tumor site, which was accompanied by macrophage and neutrophil infiltration and a significant increase in IFNγ production (50). Similarly, immunization of mice bearing a subcutaneous mammary tumor (LM3 adenocarcinoma) with attenuated *S. typhi* in the peritumoral tissue and the tumor draining lymph nodes initiated an anti-tumor Th1 response that was characterized by increased frequencies of IFNγ-secreting CD4^+^ and CD8^+^ T cells within the tumor. Treated mice displayed a reduced tumor growth and lung metastasis, and prolonged survival compared to unimmunized mice (28). In another study, when mice bearing Her2/neu-expressing CT-26 tumors were injected with a recombinant attenuated *S. typhimurium* vaccine intratumorally, a significant reduction in tumor growth was evidenced. This effect on tumor growth was partially lost upon depletion of CD8 T cells, suggesting a role for these cells in *Salmonella*-mediated tumor suppression (24). Using the attenuated *S. typhimurium* strain A1-R, which was originally derived by *in vivo* passaging through tumor tissue (51), Murakami and colleagues recently demonstrated its efficacy in promoting CD8 T cell infiltration and tumor growth arrest in a syngeneic pancreatic-cancer orthotopic mouse model (52). The immunotherapeutic effect of *L. monocytogenes* is also partially mediated by cytotoxic T lymphocytes (CTLs). Immunization of mice carrying triple negative mammary tumors (4T1 cells) with *Listeria* led to direct killing of tumor cells by the bacteria, eventually leading to the eradication of the primary tumor and all the metastases. Depletion of CD8 T cells partially restored tumor growth in these mice, indicating that CTLs also play an important role in *Listeria*-mediated tumor therapy (53). Finally, treatment of ovarian carcinoma with attenuated strain of the parasite *T. gondii* (CPS) led to an increase in CD4^+^ and CD8^+^ T cell infiltration into the tumor microenvironment, activation of tumor-resident T cells, and enhanced IFN-γ production by T cell populations (31). Taken together, these data highlight the immune-potentiating, anti-tumor effects of bacterial therapy in diverse cancer models.

Bacterial immunotherapeutic agents can induce memory T cell responses as well. Treatment of mice harboring established hepatic metastases of colorectal cancers with an attenuated strain of *L. monocytogenes* expressing a tumor-associated antigen led to a strong initial tumor specific CD8 T cell response that successfully treated 90% of the animals (54). It also generated central and effector memory T cells that protected the mice against tumor re-challenge. Additionally, the treatment led to a decrease in the expression of PD-1, an immune inhibitory molecule expressed on tumor-infiltrating lymphocytes, demonstrating the efficacy of this vaccine to down modulate the immunosuppressive tumor microenvironment (54).

### Gamma-Delta (γδ) T Cells

Gamma-delta T cells are a subset of T lymphocytes characterized by the presence of a surface antigen recognition complex type 2. Activated γδ T cells exhibit potent anti-tumor activity by releasing copious amounts of IFN-γ and TNF-α (55). Mycobacterial immunotherapy using *Mycobacterium vaccae, M. obuense*, or BCG induced the proliferation and activation of γδ T cells (56). Activated γδ T cells showed enhanced effector responses including upregulated granzyme expression and production of Th1 cytokines, such as IFN-γ and TNF-α (56). When *Listeria moncytogenes* was used as immunotherapeutic agent against cervical cancer in mice, increased levels of intratumoral IL-17 and IL-17-positive γδ T cells were observed (57). However, despite the activation of γδ T cells by *Listeria* treatment in tumor-bearing mice, tumor progression was unaltered in γδ T cell-deficient mice. Instead, *Listeria*-induced anti-tumor activity was critically dependent on αβ T cells. This indicated that, at least in this cervical cancer model, *Listeria* immunotherapy-associated γδ T cell activation is of secondary importance (57).

## Effect of Bacterial Therapy on Immunosuppressive Cells

### Myeloid-Derived Suppressor Cells

Myeloid-derived suppressor cells, the immature myeloid cells present in the bone marrow, peripheral blood, and spleen under normal physiological conditions, can undergo tremendous expansion in various pathological conditions, such as cancers and autoimmune diseases (58). Recruitment of MDSCs and Tregs into the tumor microenvironment helps tumors to evade the host immunosurveillance system. In cancers, these cells suppress CD4 and CD8 T cell-mediated anti-tumor responses by the nitration of the T cell receptors (59). Hence, MDSCs are regarded as one of the most important targets for cancer therapy. Several drugs that differentiate MDSCs into mature phenotypes are in use for cancer therapeutic purposes.

Recently, we demonstrated that treatment of B16.F1 melanoma with an attenuated strain of *S. typhimurium* leads to an increase in the CD11b^+^Gr1^+^ myeloid cells in both spleen and tumor (23). Subsequent to *Salmonella* treatment, intratumoral myeloid cells exhibited a significant loss in their immunosuppressive capacity. Interestingly, this phenotype shift was not observed among the splenic myeloid cells, suggesting that the splenic and intratumoral myeloid cells respond differentially to *Salmonella* treatment. Moreover, we observed an increase in the expression of co-stimulatory molecules, such as CD40, CD80, and CD86 on myeloid cells in the treated group of mice, suggesting that the myeloid cells shifted from an immature to mature phenotype following *Salmonella* inoculation (23). Further analysis of intratumoral MDSCs indicated that *Salmonella* treatment resulted in a significant increase in the proportion of M-MDSCs (Figure 1O). Furthermore, these cells increased their expression of the differentiation markers Sca-1 (Figures 1P,Q) and MHC class II (Figures 1R,S), indicative of their activity level and maturation status. This suggested that *Salmonella* treatment triggered the differentiation of intratumoral MDSCs, causing a reduction in their immunosuppressive properties.

Wallecha and coworkers demonstrated that cancer immunotherapy using *L. monocytogenes*-LLO (Lm-LLO) diminished the suppressive functions of MDSCs and Tregs in the tumor microenvironment (60). This diminished suppressive function was linked to a reduction in the expression of Arg1 by MDSCs and IL-10 by Tregs (60). While the former decreases the expression of the T-cell receptor CD3ς chain (19), the latter is a pleiotropic immunosuppressive cytokine (61). When used as an immunotherapeutic vaccine to treat 4T1 tumors in young and old aged mice, *L. monocytogenes*-infected MDSCs in the blood and the primary tumors causing a reduction in their number and converting them into immune-stimulating, IL12-producing macrophages. This was accompanied by a dramatic reduction in tumor metastasis and tumor growth (62). A recent study by Zhang and colleagues demonstrated the capacity of bacterial lipoprotein (BLP), a TLR1/2 agonist, to decrease intratumoral MDSC accumulation while allowing for infiltration by IFNγ-expressing CD8^+^ T cells (63). Using a syngeneic glioma mouse model, the authors showed that mice treated with a systemic injection of BLP and adoptively transferred antigen-specific T cells had improved survival. The alterations in MDSC and CD8^+^ T cells ratios within the tumor microenvironment were associated with increased expression of CXCL10, a chemokine that induces CD8^+^ T cell migration, and a reduction in the expression of CCL2, known to regulate the migration of MDSC (63).

### Regulatory T Cells

Regulatory T cells are implicated in autoimmune diseases and cancers. Under healthy conditions, CD4^+^CD25^+^Foxp3^+^ Tregs function to prevent immune reactions to autoantigens. However, these cells are known to attenuate anti-tumor immunity by inhibiting tumor antigen-specific CTLs (64). Recent studies showed that Tregs could be targets of bacterial immunotherapy against cancer. Peritumoral injection of attenuated *S. typhi* in mammary adenocarcinoma-bearing mice led to a reduction in T regulatory cells in tumor draining lymph nodes and led to decreased metastasis and enhanced overall host survival (28). Likewise, intratumoral injection of attenuated *Salmonella* reduced the percentage of CD25^+^FoxP3^+^ cells among spleen and tumor CD4^+^ T cells in a colon cancer model (24). The precise reason for the effect of *Salmonella* vaccines on Tregs is obscure. However, it is known that *Salmonella* treatment leads to the downregulation of CD44, a key cell surface molecule on Tregs as well as tumor cells, and contributes to tumor angiogenic invasiveness and proliferative potential (65). Interestingly, systemic administration of a synthetic BLP, a TLR1/TLR2 agonist, to mice with established lung carcinoma, melanoma, or leukemia, led to tumor regression and a long-lasting protective response against tumor re-challenge (66). The BLP-mediated immunotherapeutic effect was due to a reduction in the suppressive function of Tregs and a concomitant enhancement in anti-tumor CTL response (66). In another study, intraperitoneal administration of a *lppAB/msbB* mutant of *S. typhimurium* (lacking Braun lipoprotein; LPP) failed to induce anti-tumor activity against subcutaneously implanted B16.F10 tumors. This indicated that LPP is a critical factor for the anti-tumoral activity of attenuated *Salmonella* (65).

**Figure 2:**
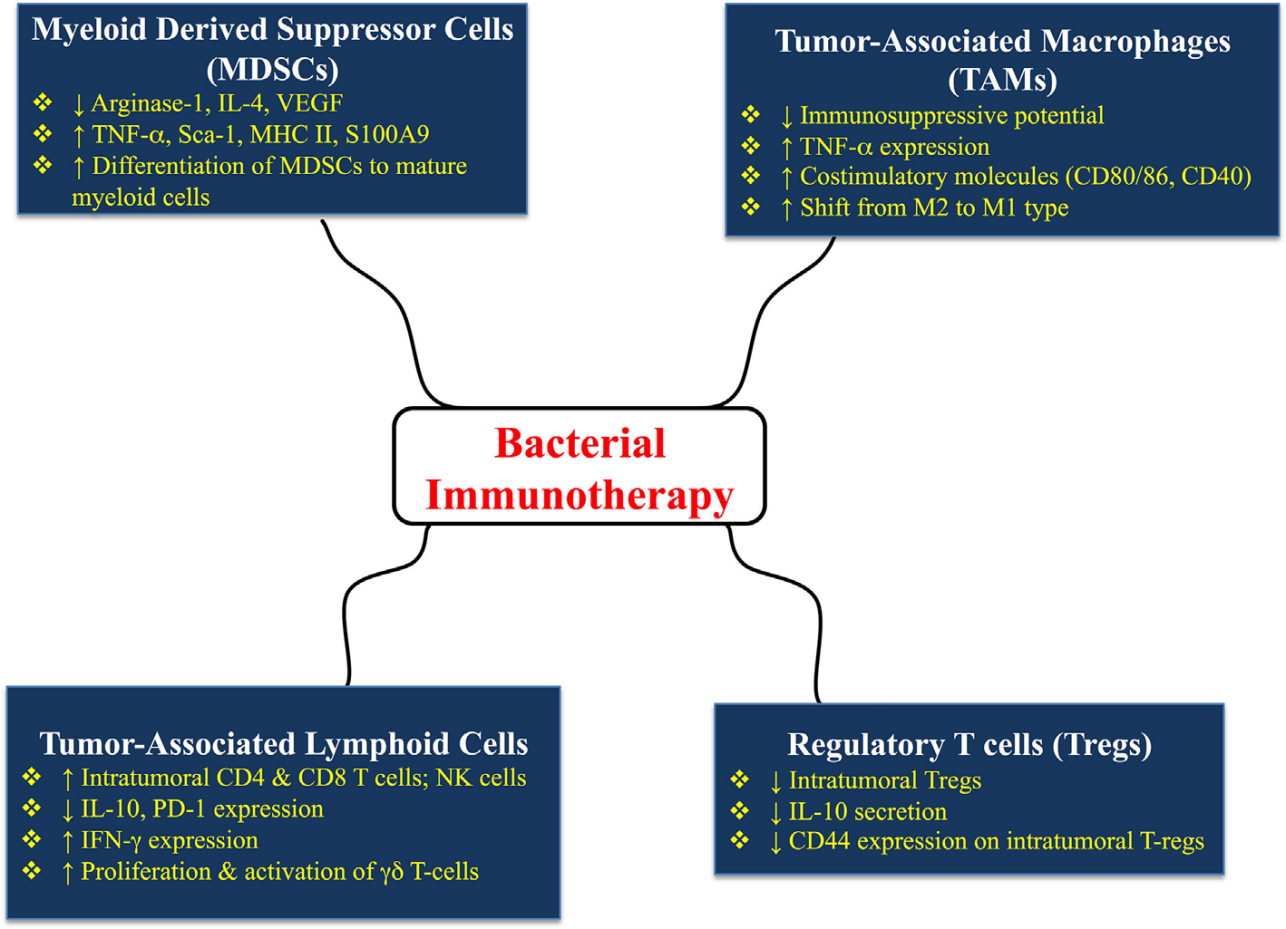
Multiple effects of bacterial immunotherapy on different immune cell types in tumor-bearing hosts. The details and references for the summarized changes are discussed in the text.

A schematic summary of the multiple effects of bacterial immunotherapy on different immune cell types is shown in Figure 2.

## Concluding Remarks

More than 120 years after Coley’s documentation of the effect of bacterial infections on human tumors, various forms of “Coley’s toxins” are currently being actively developed as therapeutic agents to treat cancers (67). The greatest challenge for a wide acceptance of this approach has been the concerns about the safety of using potentially pathogenic bacteria for therapeutic purposes. In order to overcome this limitation, genetically modified organisms have been developed in such a way to balance the requirement for safety while maintaining therapeutic efficacy. There are many examples where engineered strains of *Salmonella enterica* serovar Typhimurium, *L. monocytogenes*, and *Clostridium novyi*-NT have been used in preclinical models as well as in clinical trials (4, 68–72). Although so far limited, a few examples of the use of attenuated bacteria in clinical trials have been reported. Intratumoral injection of *C. novyi*-NT spores has been successfully used to treat a patient with advanced leiomyosarcoma (4). Moreover, the safety of attenuated *L. monocytogenes* strains has been demonstrated in patients with advanced cancers (71, 72).

Accumulating evidence has demonstrated that heavy alterations in the pathogenicity of bacteria through the introduction of mutations to alleviate safety concerns actually compromise their therapeutic potential. However, important achievements have improved this perspective. Notably, it was recently shown that mutations in genes involved in the shikimate pathway, such as *aroA*, attenuates *Salmonella* while enhancing their immunogenicity (73). Furthermore, mutations that lead to modifications in lipid A and flagella synthesis result in an increased immune-stimulatory capacity and as such the mutant strain was able to overcome the efficacy-limiting effects of pre-exposure (74). Conditional expression of LPS by using an inducible promoter has been useful in enhancing the intrinsic anti-tumor effects of attenuated *Salmonella* strains (75).

It is currently clear that using live bacteria to treat cancer is a form of immunotherapy. However, the precise mechanisms underlying this process remain incompletely understood. The findings to date point to strong immunomodulatory effects of bacterial therapy within the tumor microenvironment. This is perhaps best illustrated in the case of *Salmonella* therapy where the accumulation of bacteria within the tumor tissue leads to increased immunogenicity of tumors. Given that myeloid cells are the natural habitat for *Salmonella* organisms, these cells appear to be the major modulatory targets of this form of anti-tumor therapy. *Salmonella*-mediated inhibition of B16.F1 melanoma was not compromised when tested in nude mice, suggesting that the anti-tumor effect of *Salmonella* therapy is thymus-independent (23). Instead, effective *Salmonella* therapy was totally abrogated in mice deficient in the TLR-MyD88 signaling pathway (23), implicating innate immune cells as the primary target of this intervention. It is likely that the contribution of the different arms of the immune system to *Salmonella*-mediated cancer control is highly dependent on the relative tumorigenicity and immunogenicity of the tumor being investigated. As demonstrated by findings in our laboratory and others, successful inhibition of tumor growth by *Salmonella* is associated with the transformation of suppressive M2-like myeloid cells to inflammatory M1-type mature macrophages, which in turn likely leads to an enhancement in anti-tumor T cell responses. The capacity of *Salmonella* organisms to induce changes in macrophage functions in infections and cancer is well established (37, 76, 77). The effectiveness of anti-cancer bacterial therapy through targeting myeloid cells has refocused attention on the importance of these lymphoreticular “white” cells in tumors, first described by Virchow more than 150 years ago (78). Our increased understanding of the properties of bacteria that enhance their anti-tumor and immunostimulatory capacity, and their precise cellular and molecular targets within the tumor microenvironment, promises to usher in new modalities for cancer treatment.

## Ethics Statement

Animal studies were carried out in accordance with, and after approval of the Animal Research Ethics Committee of the CMHS (Protocols #A13/15 and A26/13).

## Author Contributions

SK performed experiments, analyzed data, and wrote the paper. AA-S contributed to the flowcytometric analysis. OC-M contributed in writing the paper. MF-C wrote the final manuscript. BA-R designed the study, supervised the project, analyzed data, and wrote the final manuscript.

## Conflict of Interest Statement

The authors declare that the research was conducted in the absence of any commercial or financial relationships that could be construed as a potential conflict of interest.
